# Modulation of intestinal epithelial cell proliferation and apoptosis by *Lactobacillus gasseri* SF1183

**DOI:** 10.1038/s41598-022-24483-0

**Published:** 2022-11-24

**Authors:** Blanda Di Luccia, Vittoria Acampora, Anella Saggese, Viola Calabrò, Maria Vivo, Tiziana Angrisano, Loredana Baccigalupi, Ezio Ricca, Alessandra Pollice

**Affiliations:** 1 Department of Biology, Federico II University, Naples, Italy; 2 Department of Molecular Medicine and Medical Biotechnology, Federico II University, Naples, Italy; 3 Department of Chemistry and Biology A. Zambelli, University of Salerno, Salerno, Italy; 4 Department of Microbiology and Immunology, Stanford University-School of Medicine, Stanford, CA USA

**Keywords:** Cell biology, Microbiology

## Abstract

The gut microbiota exerts a variety of positive effects on the intestinal homeostasis, including the production of beneficial molecules, control of the epithelial barrier integrity and the regulation of the balance between host’s cell death and proliferation. The interactions between commensal bacteria and intestinal cells are still under-investigated and is then of paramount importance to address such interactions at the molecular and cellular levels. We report an in vitro analysis of the effects of molecules secreted by *Lactobacillus gasseri* SF1183 on HCT116 cells, selected as a model of intestinal epithelial cells. SF1183 is a *L. gasseri* strain isolated from an ileal biopsy of a human healthy volunteer, able to prevent colitis symptoms in vivo. Expanding previous findings, we show that bioactive molecules secreted by SF1183 reduce the proliferation of HCT116 cells in a reversible manner determining a variation in cell cycle markers (p21WAF, p53, cyclin D1) and resulting in the protection of HCT116 cells from TNF-alfa induced apoptosis, an effect potentially relevant for the protection of the epithelial barrier integrity and reconstitution of tissue homeostasis. Consistently, SF1183 secreted molecules increase the recruitment of occludin, a major component of TJ, at the cell–cell contacts, suggesting a reinforcement of the barrier function.

## Introduction

The intestinal microbiota co-evolved with its animal host forming a symbiotic relationship and contributing to the metabolic health of the host. The relative distribution of gut bacteria is unique to an individual and changes transiently depending on diet, age, body exercise, drug usage and host genetics, making it difficult to define a normal or healthy microbiota. However, a high taxa diversity, high richness and stable microbiota functional cores characterize healthy gut microbial communities^1^. Drastic alterations of such microbial composition (dysbiosis) have been associated to the pathogenesis of various metabolic disorders and intestinal inflammatory diseases, including colon inflammations (UC, Ulcerative Colitis, CD, Chron Disease), generally referred as Inflammatory Bowel Diseases (IBDs). IBDs are characterized by an abnormal production of inflammatory cytokines (TNF-α, Tumor Necrosis Factor alpha, IFN-γ Interferon Gamma) by host cells and disruption of the epithelial integrity in part due to enhanced apoptosis. In order to balance these conditions, a series of microbiota-directed intervention strategies have been proposed and tested in animal models and, in some cases, in human trials. These include the oral administration of (1) prebiotics, undigestible oligosaccharides (for example, inulin and oligofructose) that, once fermented by some members of the microbiota, cause an improvement of gut barrier functions, (2) probiotics, live bacteria that, colonizing the gut exert a beneficial health effect (3) synbiotics, a combination of prebiotics and various probiotic strains; and (4) postbiotics, pasteurized probiotics or parts of microbial strains^1^.

The oral use of probiotics is currently very well accepted because of the capacity of some bacteria to influence cytokine expression, reduce inflammation and protect gut barrier integrity^2–6^. The use of probiotics originated from traditional foods containing live bacteria, common in many cultures and considered to have health beneficial effects. Well-known examples in this context are lactic acid bacteria, present in a variety of traditional fermented foods^7^ and spore formers of the *Bacillus* genus found in Natto, a Japanese traditional healthy food based on soybeans fermented with the *B. subtilis* var. *natto* and in similar fermented foods traditionally used in various countries^8^. Several species of the *Bifidobacterium, Lactobacillus*, *Bacillus* and *Saccharomyces* genera have long been used as commercial probiotics^8,9^. In addition, other intestinal bacteria have been proposed as next-generation probiotics, such as *Faecalibacterium prausnitzii*^10^ and *Akkermansia muciniphila*^11^.

The molecular mechanisms controlling the interaction between intestinal cells and bacteria have been studied in vitro in some cases. Examples are *L. casei Shirota* that has immunomodulatory effects by inducing Interleukin-12 (IL-12) production; *L. fermentum* NCIMB 5221 that has an antiproliferative effect and modulates hyperinsulinemia, insulin resistance, hypercholesterolemia, and hypertriglyceridemia; *Saccharomyces cerevisiae* var.*boulardii* that has anti-inflammatory and antibacterial effects by increasing the secretion of immunoglobulin A (IgA) and maintaining the integrity of the epithelial barrier^12^. Often, quorum-sensing autoinducers, communication molecules released by bacteria at high densities, have been shown to modulate host responses either directly or through regulation of bacterial genes. Examples in this context are quorum-sensing peptides of various *Bacillus* species that are taken up by intestinal cells and contribute to eukaryotic cell homeostasis activating survival pathways (p38 MitogenActivatedProteinKinase (MAPK) and protein kinase B)^13,14^. In some cases, the secreted bacterial effectors have not been identified: molecules secreted by *L. reuteri* were found to potentiate tumour necrosis factor (TNFα)-induced apoptosis in myeloid leukemia-derived cells by suppressing Nuclear Factor-kB (NF-kB) activation by inhibiting IkBa degradation, downregulating nuclear factor-kB (NF-kB)-dependent gene products and promoting apoptosis by enhancing MAPK activities including c-Jun N-terminal kinase and p38 MAPK^15^.

In this context, our study investigates the in vitro effects of bioactive molecule/s produced and secreted by a strain of *L. gasseri* on colorectal HCT116 cells, both at the molecular and cellular levels. *L. gasseri* is one of the major homofermentative *Lactobacillus* species of the human intestine and strain SF1183 was isolated from an ileal biopsy of a human healthy volunteer^16^. In particular, it was isolated from a sub-population of bacteria tightly associated to the epithelial cells and was shown to have antimicrobial activity against a panel of entero- pathogens and to form a biofilm in standard laboratory as well as in simulated intestinal conditions^16^. Our previous in vivo study showed that SF1183 has protective effects on the intestinal epithelium, reinforcing the tight junctions and preventing Dextran-Sulfate-Sodium-induced (DSS)-induced colitis symptoms without major effects on the overall microbial composition of the gut^17^.

We here provide further evidences that *L. gasseri* SF1183 is able to influence HCT116 homeostasis by affecting cell proliferation in a reversible manner, reducing TNF-α induced apoptosis and increasing occludin recruitment at the cell periphery. We characterized the effect at the molecular and cellular level showing alterations of cell proliferation markers and changes in the cell shape strongly indicating a reinforcement of the barrier functions.

## Results

### The conditioned medium of *L. gasseri* reduces TNF-α induced apoptosis of HCT116 cells

**Figure 1:**
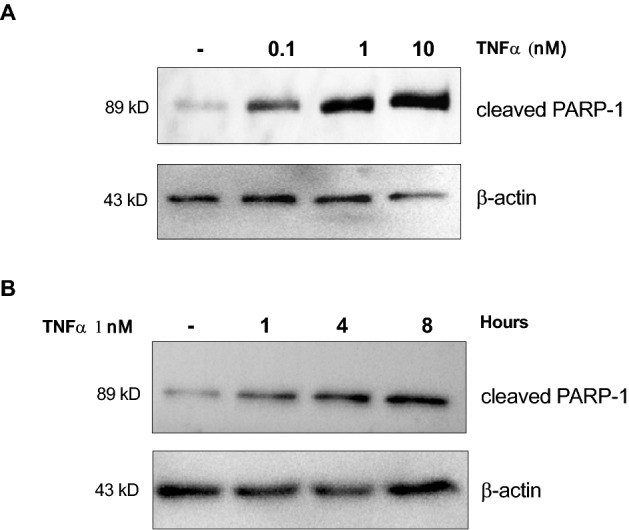
HCT116 cells respond to TNF-α induced apoptosis (**A**) HCT116 cells were incubated with TNF-α at the concentrations indicated for 8 h. Cells were collected and whole protein extracts were analyzed by western blot with antibodies against cleaved PARP-1 and β-actin. (**B**) HCT116 cells were incubated with TNF-α 1 nM for 1, 4 or 8 h. Cells were collected, and whole protein extracts were analyzed by western blot with antibodies against cleaved PARP-1 and β-actin.

To study the effects of molecules secreted by *L. gasseri* SF1183 on cytokine-induced apoptosis, the HCT116 intestinal epithelial cell line was selected for its sensitivity to TNF-α^18^. To set up the experimental conditions, different concentrations of TNF-α and different incubation times were used to treat HCT116 cells (Fig. 1). The induction of the apoptotic program was monitored through the analysis of the proteolytic cleavage of PARP-1 (Poly ADP-ribose polymerase 1); in particular, the detection, by western blot, of PARP-1 (89 kDa) catalytic subunit resulting from Caspase 3 activity was used as an apoptotic marker in our experimental setting. As expected, HCT116 cells responded to TNFα in a dosage and time-dependent manner (Fig. 1). The conditions of 1 nM TNF-α for 8 h of incubation was chosen for subsequent experiments since it determined a partial PARP-1 cleavage therefore allowing the detection of increased or decreased levels of apoptosis.

**Figure 2:**
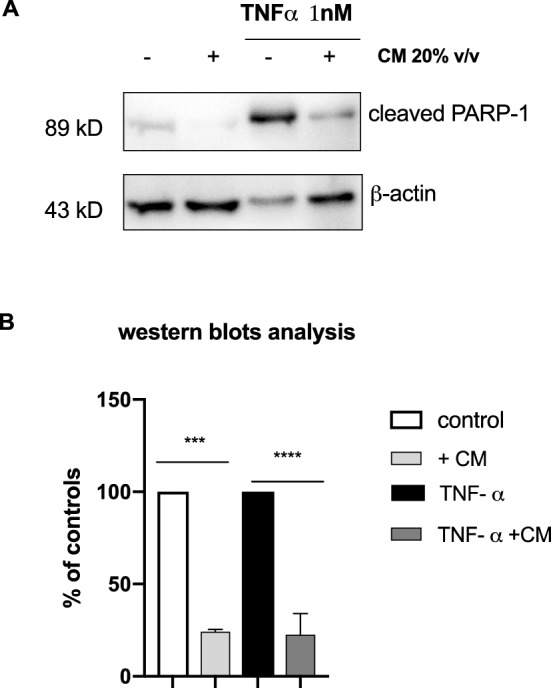
The conditioned Medium of *L. gasseri* SF1183 reduces apoptosis of HCT116 cells. (**A**) HCT116 cells were incubated or not with CM for 16 h where indicated; after that, cells were incubated with TNF-α 1 nM for additional 8 h where indicated. Cells were collected and extracts were analyzed by western blot with antibodies against cleaved PARP-1 and β-actin. (**B**) Quantification of normalized cleaved PARP-1 levels from three independent replicates performed by ImageLab. Statistical analyses were performed by unpaired t-test. Levels of significance are indicated (***p = 0.0002, **** p < 0.0001).

To obtain the conditioned medium (CM) for subsequent analysis, *L. gasseri* SF1183 was grown up to the stationary phase, the medium was filter sterilized and then added (20% v/v) to HCT116 cells for 16 h. After that, cells were incubated (Fig. 2, lanes 2 and 4) or not (Fig. 2, lanes 1 and 3) with 1 nM TNF-α for 8 h without removing the CM. At the end of the incubation time, cells were collected, whole cell protein extracts were prepared and analyzed by western blotting with antibodies specific for the cleaved 89 kDa PARP-1 (Cell Signaling). As shown in Fig. 2, the proteolytic cleavage of PARP-1 (that is detectable in untreated cells, see lane 1) was increased by the TNF-α treatment (lanes 1 vs 3) and strongly reduced in presence of CM both in cells treated or not with TNF-α (lanes 1 vs. 2 and 3 vs. 4; p = 0.0002, p < 0.0001). Reduction of apoptosis in cells not treated with TNF-α is noteworthy and suggests an anti-apoptotic effect of molecules present in the CM of *L. gasseri* also under physiological conditions of growth in which apoptosis occurs at a basal level (Fig. 2, lanes 1 vs. 2). No reduction of apoptosis was observed when HCT116 cells were treated with fresh bacterial medium acidified to pH 4.0 with lactic acid, ruling out the possibility that the effect observed with the CM was due to the acidification of the medium caused by the fermentative growth of *L. gasseri* (data not shown).

The anti-apoptotic effect exerted by the CM was due to proteinaceous molecule(s) smaller than 3 kDa whose characterization is currently in progress (Supplementary Fig. 1).

### The conditioned medium of *L. gasseri* affects proliferation of HCT116 cells in a reversible manner

**Figure 3:**
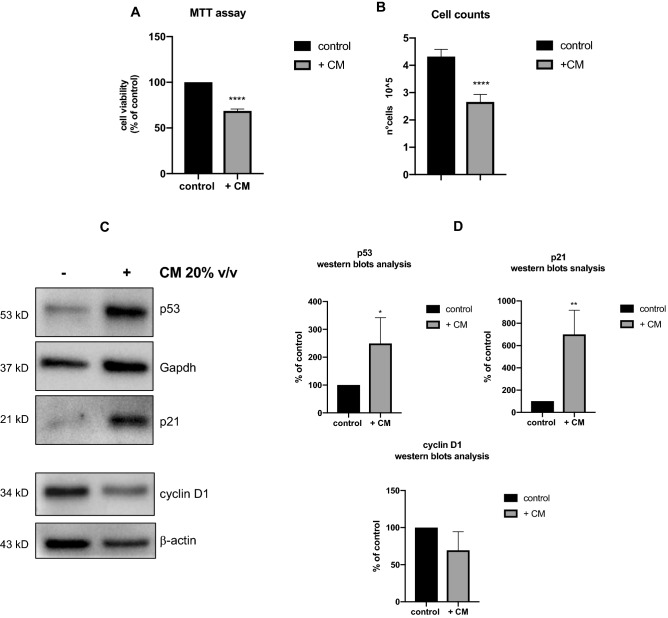
The conditioned Medium of *L. gasseri* SF1183 reduces proliferation of HCT116 cells. (**A**) Proliferating HCT116 cells were incubated in complete cell culture media supplemented or not with CM (20%v/v) for 16 h; after that, cells were washed out from CM and treated for MTT assay. Statistical analyses were performed using unpaired t-test. Levels of significance between points of expression are indicated (**** p < 0.0001). (**B**) Proliferating HCT116 cells were incubated in complete cell culture media supplemented or not with CM (20%v/v) for 16 h; after that, cells were washed out from CM After 24 h cells were collected and counted. Levels of significance between points of expression are indicated (**** p < 0.0001). (**C**) Proliferating HCT116 cells were incubated for 16 h in complete cell culture media supplemented with CM where indicated; after that, cells were collected, whole cell extracts were prepared and analyzed by western blot with antibodies against p53, p21, cyclinD1, b-actin and Gapdh. (**D**) Quantification of normalized protein levels from three independent replicates performed by ImageLab Statistical analyses were performed using unpaired t-test. Levels of significance between points of expression are indicated (* p = 0.00482; ** p = 0.0088).

To characterize the effects exerted by the CM of *L. gasseri* SF1183 on HCT116 cells, we performed an MTT assay to analyze the viability of HCT116 cells grown for 24 h in the presence of CM. As shown in Fig. 3A, the CM caused a 30% reduction of cell viability (p < 0.0001). A similar reduction was observed with the evaluation of the HCT116 cell number measured in the same conditions used for the MTT experiment (Fig. 3B; p < 0.0001), thus suggesting that the reduction of viability was due to a reduction in the number of cells and not of their metabolic activity, allowing us to hypothesize that the CM of *L. gasseri* contains molecules capable of inhibiting the proliferation of HCT116 cells. To verify such hypothesis, western blot analyses of biomarkers of cell proliferation were performed. We observed a drastic induction of cell cycle arrest markers p53 and p21WAF (increased about 2.5 and 8 folds, respectively; 0.00482; p = 0.0088) and a reduction of a cell proliferation marker (cyclin D1) in cells incubated with CM for 24 h (Fig. 3), fully supporting our hypothesis.

**Figure 4:**
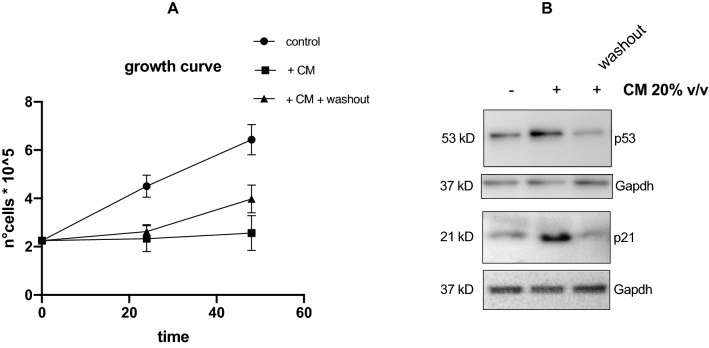
The conditioned Medium of *L. gasseri* SF1183 affects HCT116 proliferation in a reversible manner. (**A**) Growth curve of HCT116 cells either grown in complete medium (control, circles) or in medium supplemented with CM (squares). Cells were also grown for 16 h in presence of CM; after that, cells were washed out from CM and let grow till 24 h and 48 h total (curve with triangles). Statistical analyses were performed using 2-way ANOVA and Turkey’s multiple comparison test. Levels of significance (**** p < 0.0001). (**B**) At 24 h of growth an aliquot of cells was collected and whole protein extracts were analyzed by western blot with antibodies against p53, p21 and Gapdh. Band intensities were normalized on Gapdh of each blot and expressed as ratio respect to control.

The negative effect of the CM on the proliferation of HCT116 cells was partially reversible. As shown in Fig. 4A, HCT116 cells grown in presence of CM for 16 h experienced a cell proliferation arrest (closed squares in Fig. 4A) that was partially reversed when the CM was removed, the cells washed and resuspended in fresh medium (closed triangles in Fig. 4A) (p < 0.0001). The partial recovery of cell growth upon CM removal, was confirmed by the reduction of the intracellular levels of p53 and p21WAF (Fig. 4B) induced by CM treatment. Altogether, results of Fig. 4 indicate that the CM of *L. gasseri* SF1183 has a reversible effect on HCT116 cell proliferation.

### The conditioned medium of *L. gasseri* affects occludin localization in HCT116 intestinal epithelial cells

**Figure 5:**
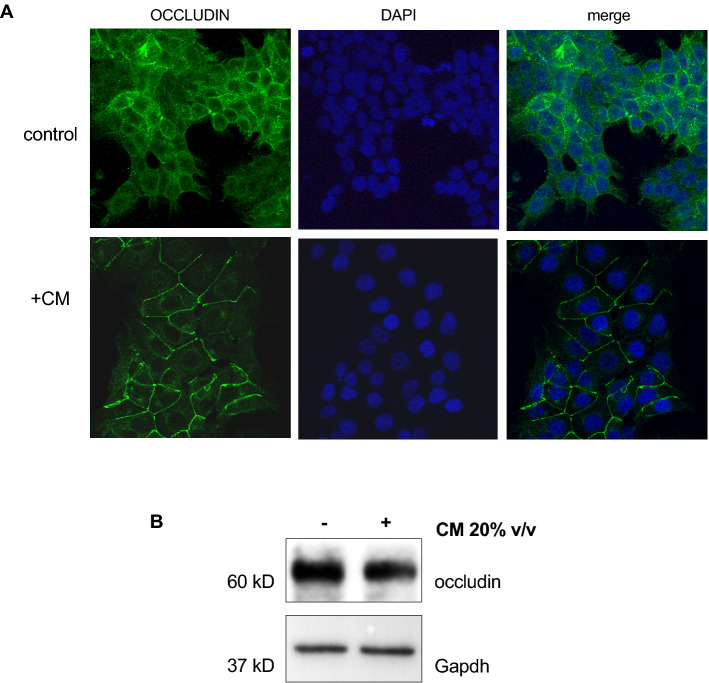
The conditioned Medium of *L. gasseri* SF1183 affects HCT116 exerts a wider protective effect on HCT116 cells. (**A**) HCT116 cells were seeded onto coverslips and treated with CM for 16 h where indicated. Cells were fixed and analyzed by IF with anti-occludin, followed by DAPI to counter stain the nuclei. Representative images were taken with Zeiss confocal microscope. Scale bar 5 μm. (**B**) HCT116 cells were incubated with CM 20%v/v for 16 h. Cells were collected and extracts were analyzed by western blot with antibodies against occludin and Gapdh.

Cytoskeletal elements influence the formation of cell–cell and cell–substrate adhesions thus playing a crucial role in the formation of a healthy gastrointestinal barrier whose functions are related not only to nutrients absorption but also to protection from pathogens and inflammation. To properly function, the intestinal epithelium requires to be constituted by an intact layer of cells and junctions that seal the space between adjacent cells. It has been previously reported that *L. gasseri* SF1183 in vivo acts on tight junctions^17^. Therefore, we decided to characterize the potential barrier function exerted by CM analyzing the recruitment of occludin to cell–cell contacts. Occludin is an integral membrane protein enriched at tight junctions involved in the maintenance of structural integrity and the barrier functions. We performed immunofluorescence (IF) experiments upon CM incubation (Fig. 5). Cells were allowed to adhere onto coverslips overnight, treated with CM for 16 h as usual, fixed, and subjected to IF with anti-occludin, followed by DAPI to counter stain the nuclei. We interestingly observed a clear recruitment of occludin to cell–cell contacts of treated cells (Fig. 5 A compare upper and lower panels). Interestingly, a drastic modification of cell morphology becomes evident upon treatment, furtherly suggesting a reinforcement of cell–cell tight junctions. Remarkably, as shown in Fig. 5B, total occludin protein level did not increase upon CM treatment, thus supporting the hypothesis that molecules secreted during *L. gasseri* growth induce reinforcement of barrier functions caused by occludin relocalization to the cell periphery. This observation led to the hypothesis that the apoptosis protection could be due to the reinforcement of barrier functions caused by molecules secreted during *L. gasseri* growth.

## Discussion

The balance between cell death and proliferation is among the most important homeostatic mechanisms involved in tissues’ health, especially for those characterized by a high rate of cell renewal such as the intestinal epithelium. In this regard, probiotic bacteria, such as *Lactobacillus* and *Bifidobacterium* genera play significant roles, including the modulation of inflammation, the reduction of the oxidative stress and the control of cell proliferation^19–23^. Particularly intriguing is the involvement of commensal bacteria in intestinal inflammatory diseases, known as IBDs (Inflammatory Bowel Disease), including UC (Ulcerative Colitis) and CD (Chron Diseases). In some cases, probiotics have been shown to cause remission of Inflammatory diseases also by influencing the levels of inflammatory cytokines, thus exerting a protective effect on the gut barrier^2–6,23^ In fact, a common feature of intestinal inflammations, is the increase in inflammatory cytokines that, in turn, causes apoptosis of epithelial cells with disruption of the gut barrier. On the other hand, loss of the epithelial integrity causes the infiltration of undigested food, toxic substances, bacterial molecules and microbes in the submucous layer, further increasing local inflammation and also systemic effects. Among the most largely produced cytokines in these conditions is the proinflammatory tumor necrosis factor alpha, TNF-α, known to play a major role in controlling the balance between cell proliferation and apoptosis. Depending on the cellular conditions, TNF-α is able to mediate cell survival or cell death^24^. Pharmacological intervention with anti-TNFα treatments (corticosteroids and/or monoclonal antibodies) constitute potential therapeutic approach in IBDs^24^, although some level of toxicity can be observed. In some cases, probiotics have been used alone^25^ or in combination with antibodies to TNF-α to treat intestinal inflammation^26^. Our observation that a strain of *L. gasseri* SF1183 is able to protect intestinal epithelial cells from TNF-α induced apoptosis is in line with these approaches. It has to be noted that this strain is derived from the intestine of a healthy individual underlying its benefit to intestine; on the other hand, *L. gasseri* is a common probiotic species with known effects on human health^27^. Further, SF1183 strain was shown to be able to exert protective effect in a murine model of DSS (Dextran-Sulfate-Sodium)-induced colitis reinforcing the integrity of intestinal barrier without altering the composition of intestinal microflora^17^. We add further evidences on the beneficial effects of the SF1183 strain giving insights at the cellular and molecular levels. Molecules secreted by *L. gasseri* inhibit cell proliferation in a reversible manner by acting on the p53/p21WAF pathway. Although the precise molecular mechanisms are not known, the intracellular increase in p21WAF, a very well-known regulator of cell cycle arrest^28^ could represent the final effector of the CM effect on HCT116 cells. On the other hand, p21WAF is also considered an antiapoptotic marker appearing to play pivotal roles as a modulator of apoptosis by diverse cellular mechanisms^29^. Preliminary results on the potentially bioactive molecules responsible in the CM indicate the presence of molecules of proteinaceous nature, smaller than 3 kDa. Such metabolite(s), once identified and isolated, may have direct beneficial health effects and find application as postbiotics^30^.

Interestingly, HCT116 cells treated with CM show a drastic change in cell shape with a clear re-localization of occludin at the cell periphery, indicative of a reinforcement of cell–cell tight junctions. Previous in vivo evidences are in line with this observation considering that oral administration of *L.gasseri* SF1183 to DSS treated mice show a clear staining of occludin on the epithelial surface similar to the untreated control, indicative of a reconstituted epithelium^17^. Although our analyses have been conducted in completely different conditions (on cultured human cells incubated with *L. gasseri* conditioned medium, i.e. with secreted potentially bioactive molecules), our current results are consistent with those previously obtained in vivo. Interestingly, in our conditions, incubation with CM does not cause any increase in the occludin levels but, instead, a re-localization to cell–cell contacts.

The molecular nature of secreted molecules as well as details on the molecular pathways involved in the observed effects will be future challenging tasks. All these observations, together with the reversibility of the effects exerted on human cells, strongly indicate *L. gasseri* SF1183 as a safe and promising therapeutic tool to ameliorate intestinal inflammatory conditions.

## Methods

### Bacterial strains; preparation of the conditioned medium (CM)

The SF1183 *Lactobacillus gasseri* strain was grown in MRS broth (Difco, Detroit, Mi, USA) for 24 h at 37 °C and the culture diluted was inoculated in minimal defined medium (MDM; Glucose 10 g/L, Sodium acetate 5 g/L, KH2PO4 3 g/L, K2HPO4 3 g/L, MgSO4 *7H_2_O 0.2 g/L, l-Alanine 100 mg/L, l-Arginine 100 mg/L, l-Aspartic acid 200 mg/L, l-Cysteine200 mg/L, l-Glutamic 200 mg/L, l-Histidine 100 mg/L, l-Isoleucine 100 mg/L, l-Leucine 100 mg/L, l-Lysine 100 mg/L, l-Methionine 100 mg/L, l-Phenylalanine 100 mg/L, l-Serine 100 mg/L, l-Tryptophan100 mg/L, l-Tyrosine 100 mg/L, l-Valine100 mg/L, Nicotinic acid 1 mg/L, Pantothenic acid1 mg/L, Pyridoxal 2 mg/L, Riboflavin 1 mg/L, Cyanocobalamin 1 mg/L, Adenine 10 mg/L, Guanine 10 mg/L, Uracil 10 mg/L). Cells of SF1183 were then grown anaerobically for 48 h at 37 °C. The culture was centrifuged (5000 g for 10 min at room temperature (RT)) and the supernatant (conditioned medium, CM) was filtered-sterilized through a 0.22 μm low-protein binding filter (Millipore, Bedford, MA, USA).

### Cell cultures and treatments

Human colon HCT116 (ATCC CCL-247) were a gift of Prof. Marina De Rosa, were routinely cultured at 37 °C under 50% confluence in a humidified 5% CO_2_ incubator in RPMI-1640 (Euroclone) supplemented with 10%(v/v) FBS (Euroclone), 1% penicillin–streptomycin (Euroclone), 1% l-glutamine (Euroclone). The bacterial CM was tested at 10% and 20% v/v concentration in complete RPMI growth medium for 16 h. The latter concentration gave clearer results in reducing PARP-1 cleavage and was selected for all further experiments; MDM (bacterial medium of growth) was used at 20% v/v concentration in complete growth medium for control samples. Where indicated, TNF-α (1 nM) (Millipore, Milan, Italy) was added to the cells without removing the CM and cells harvested after 8 h of treatment.

### MTT assay, cell proliferation

Cell viability was assessed using the MTT assay (Sigma-Aldrich). It is based on the reduction of the tetrazolium ring of 3-(4,5-dimethylthiazol-2-yl)-2,5-diphenyltetrazolium bromide (MTT) by mitochondrial dehydrogenases, yielding a purple dye (formazan), which can be measured spectrophotometrically; the amount of formazan produced is proportional to the number of viable cells^31^. HCT116 cells were seeded in 96-well plates (9 × 10^3^ cells/well). Cells were then treated with 20% v/v CM as described and incubated for 3 h at 37 °C with a 1× MTT solution diluted in DMEM without Phenol Red; supernatant was removed, and acidic isopropanol 0.01 N was added to each well to dissolve insoluble formazan crystals formed. The absorbance of the samples was measured at a 570 nm using a microplate reader (Multiskan spectrum, Thermo)^32^.

For cell proliferation analysis, HCT116 cells were seeded in six-well plates at a density of 2.5 × 10^5^ cells/well and incubated for 24 h in the presence of 20% v/v. After 24 h incubation cells were collected and the number of cells in each experimental point was counted with the Scepter-Millipore counter (Handheld Automated Cell Counter).

### Immunoblotting, immunofluorescence and antibodies

For western blot analysis cells were were harvested in lysis buffer and processed as described^33^. Proteins were then transferred to a polyvinylidene difluoride membrane (PVDF, Millipore) using a Mini trans-blot apparatus (Bio-Rad) according to the manufacturer’s instructions. The membrane was then incubated with indicated antibodies. Primary antibodies were anti-rabbit cleaved PARP-1 (Cell signaling EuroClone, Milan, Italy 95415-S), anti-rabbit p21WAF1 (Thermo Fisher, Invitrogen, Thermo Fisher Italy 14-671581), anti-rabbit cyclin D1 (SP4, Invitrogen, Thermo Fisher Italy, MA5-16356) anti-mouse β-actin (C4 Santa-Cruz Biotechnology DBA Milan, Italy SC-47778), anti-mouse Gapdh (6C5 Santa-Cruz Biotechnology DBA Milan, Italy, SC-32233), anti-mouse p53 (Sigma Aldrich Merck Millipore Milan MABE327). Secondary antibodies were anti-rabbit HRP (Sigma Aldrich Merck Millipore Milan Italy 12-348) and anti-mouse (Sigma Aldrich Merck Millipore Milan Italy A9044). Proteins were visualized by enhanced chemiluminescence (ECL, Bio-Rad) and revealed by Quantity One software of ChemiDoc TM XRS system (Bio-Rad). Band intensities were quantified by ImageLab BioRad software, normalized respect to loading controls and reported as fold increase/reduction with respect to the control sample. Representative experiments are shown for each blot.

Original blots were cropped to eliminate unnecessary samples and are shown as Raw Data in Supplementary Information.

For IF experiments, cells were plated in 24 well plates at 10^5^ cells/well, treated with CM 20% v/v for 16 h and treated as described in^34–36^. Briefly, cells were fixed with Paraformaldehyde (PFA) 3.7% and incubated with anti-occludin antibodies (Invitrogen Thermo Fisher OC-3F10) 1:250 in PBS 1× Tween 0.05% followed by anti-mouse Cy3 conjugated secondary antibody (Invitrogen Thermo Fisher Alexa Fluor dye 488) 1:500 in PBS 1× Tween 0.05%. Images were taken with a Zeiss (Oberkochen, Germany) confocal laser-scanning microscope Axio Observer. A 40× objective was used, and image analysis was performed using ImageJ^34^.

## Statistical analysis

All data are expressed as means of at least three biological replicates ± standard errors (SE). The analysis of variance was carried out by using One-way ANOVA or by two-tail unpaired t-test. The statistical analysis was performed with the use of Graph-Pad Prism (Graph-Pad Software).

## Supplementary Information

Supplementary Information 1.

Supplementary Figure 1.

## Data Availability

The data presented in this study are available in the main text, figures and Supplementary Material.
