# Supplementary material for: Modulation of intestinal epithelial cell proliferation and apoptosis by Lactobacillus gasseri SF1183
Source: Sci Rep. 2022 Nov 24;12:20248. doi: 10.1038/s41598-022-24483-0 (PMC9691729; doi:10.1038/s41598-022-24483-0)
Supplement: Supplementary file 1 — Supplementary Information 1. [file 41598_2022_24483_MOESM1_ESM.pdf]

RAW DATA
SUPPLEMENTARY INFORMATIONS

Modulation of intestinal epithelial cell proliferation and apoptosis by Lactobacillus gasseri SF1183

Blanda Di Luccia°#, Vittoria Acampora°, Anella Saggese°, Viola Calabrò°, Maria Vivo°^, Tiziana Angrisano°, Loredana Baccigalupi°°, Ezio Ricca°*, Alessandra Pollice °*

°Department of Biology and °°Department of Molecular Medicine and Medical Biotechnology, Federico II University, Naples, Italy

^Department of Chemistry and Biology A. Zambelli, University of Salerno, Salerno, Italy

#Department of Microbiology and Immunology, Stanford University- School of Medicine, Stanford, CA, USA

*** corresponding authors:**
apollice@unina.it
ericca@unina.it

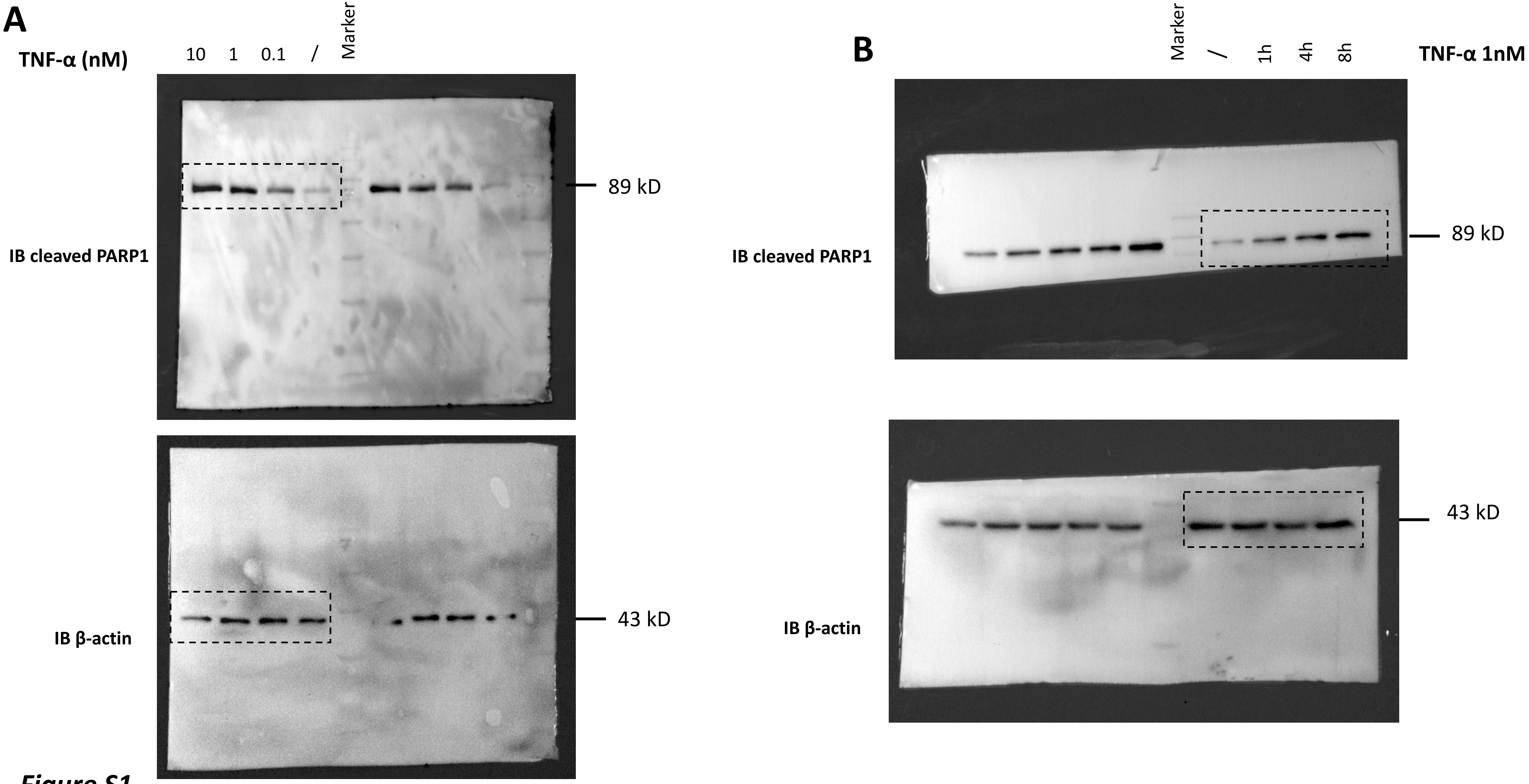

Figure S1

Full-length western blots images of cropped immunoblot of **Figure 1A and B**. Dashed squares correspond to the bands showed in the ultimate panels in the manuscript. In A bands were also horizzontally flipped.

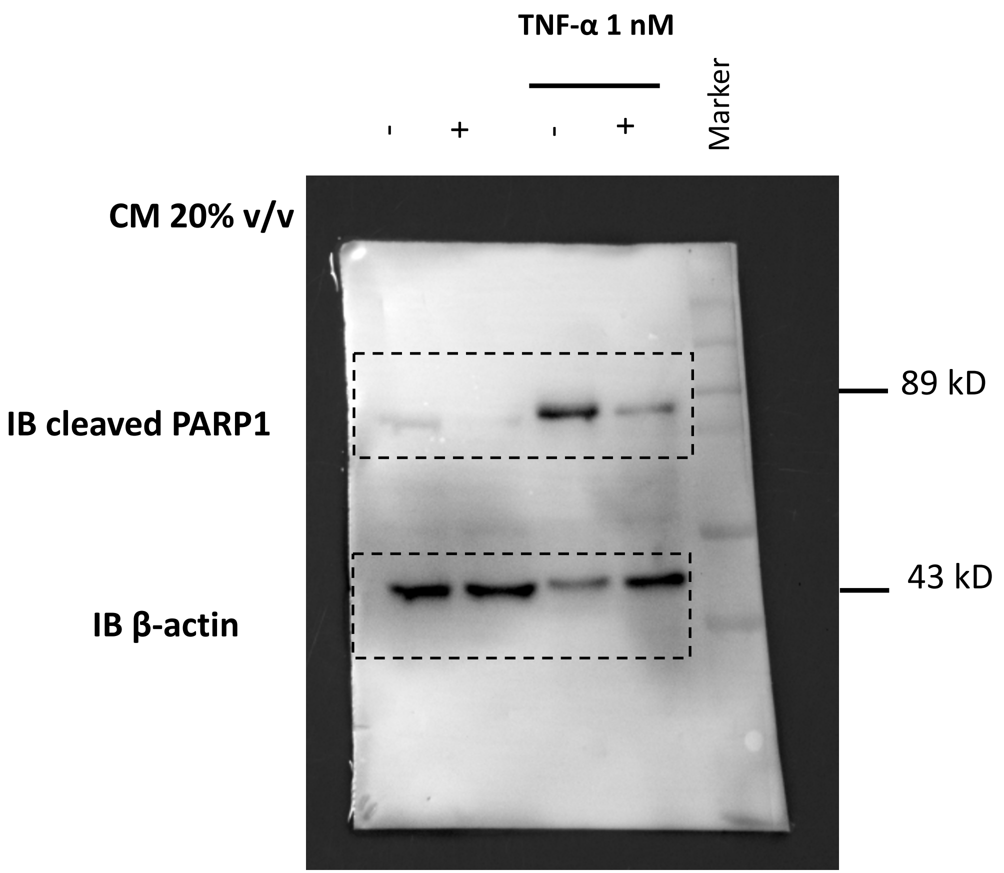

Figure S2

Full-length western blots images of cropped immunoblot of F**igure 2A**. Dashed squares correspond to the bands showed in the ultimate panels in the manuscript.

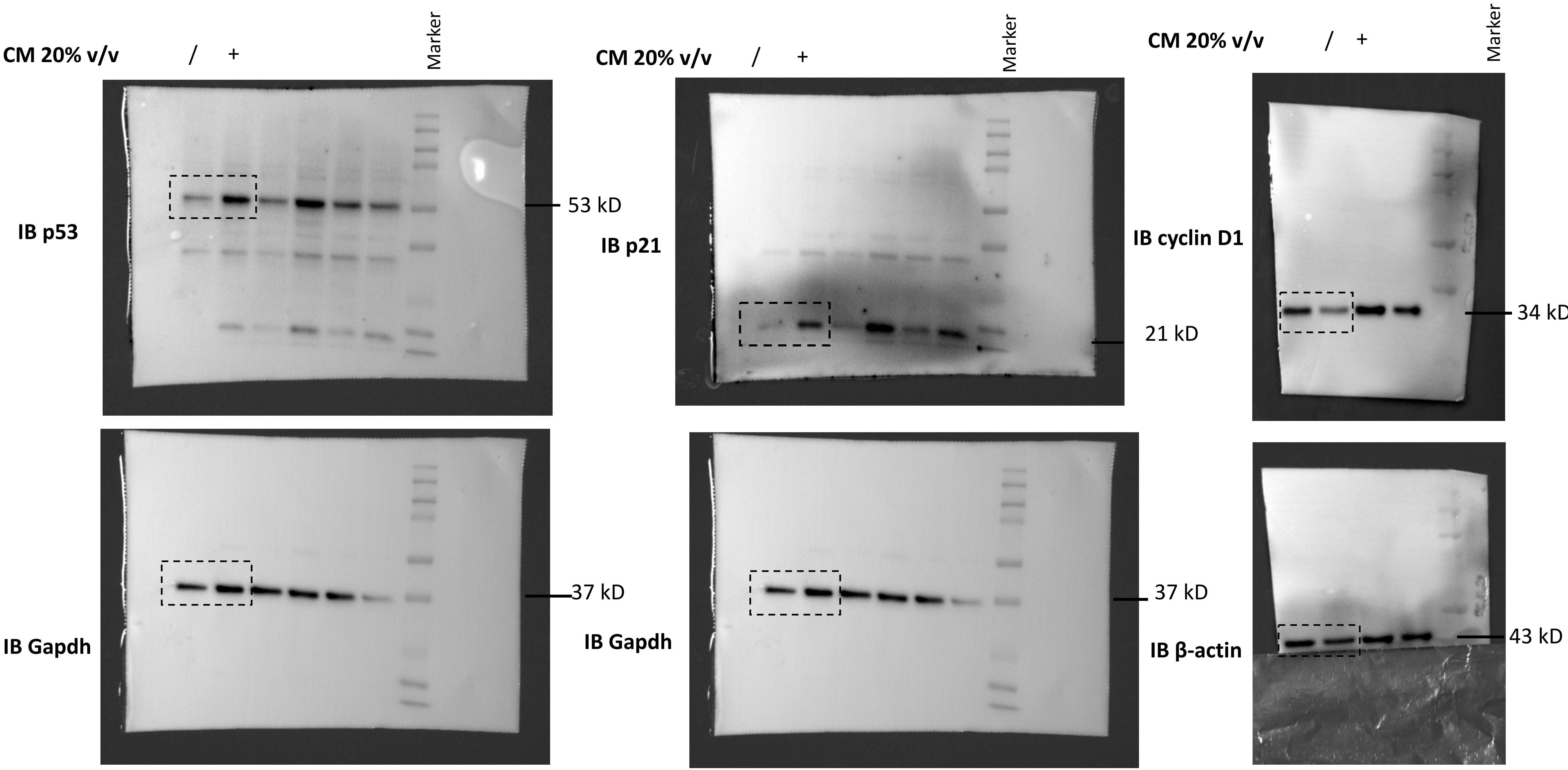

Figure S3

Full-length western blots images of cropped immunoblot of **Figure 3C**. Dashed squares correspond to the bands showed in the ultimate panels in the manuscript.

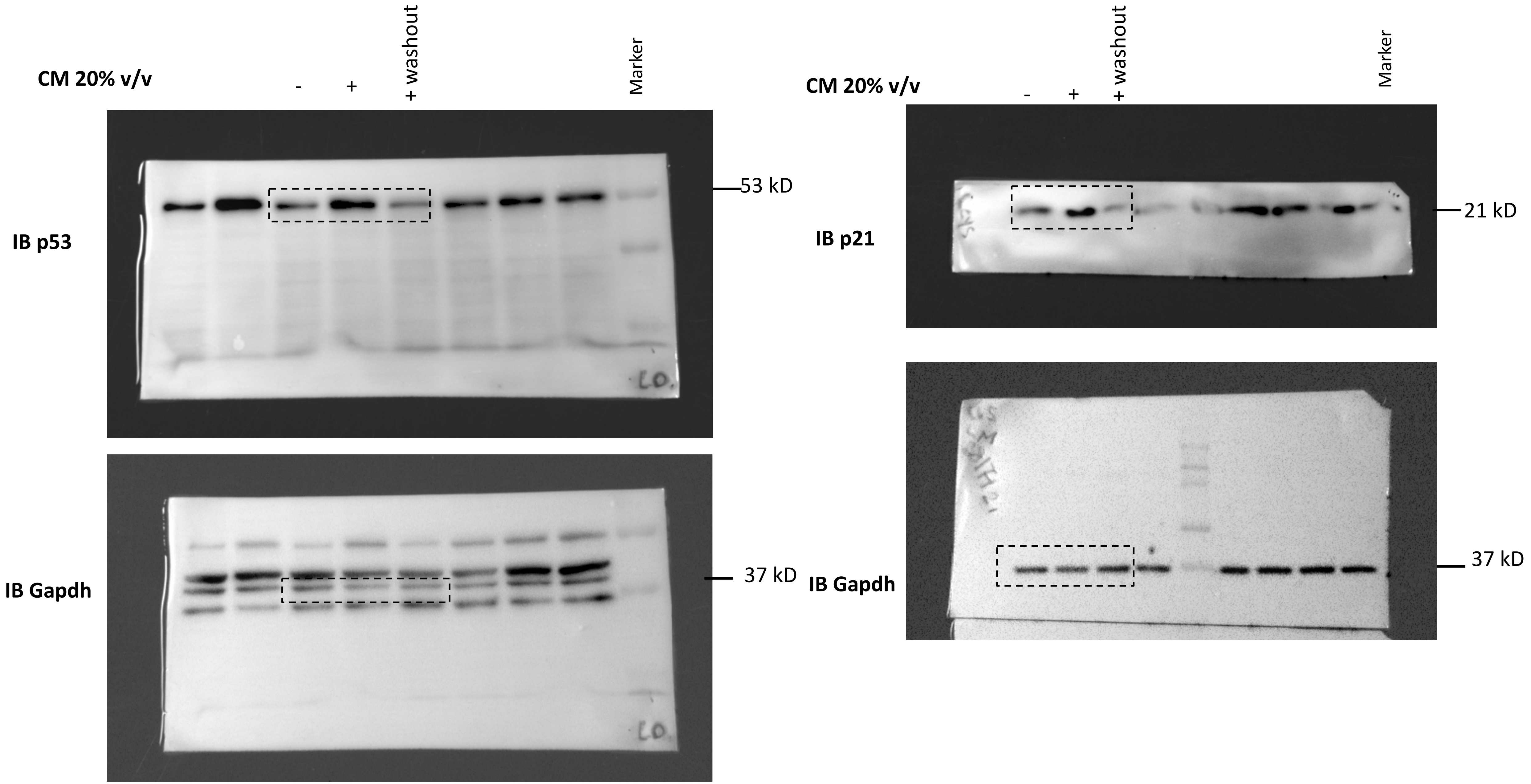

Figure S4

Full-length western blots images of cropped immunoblot of **Figure 4B**. Dashed squares correspond to the bands showed in the ultimate panels in the manuscript.

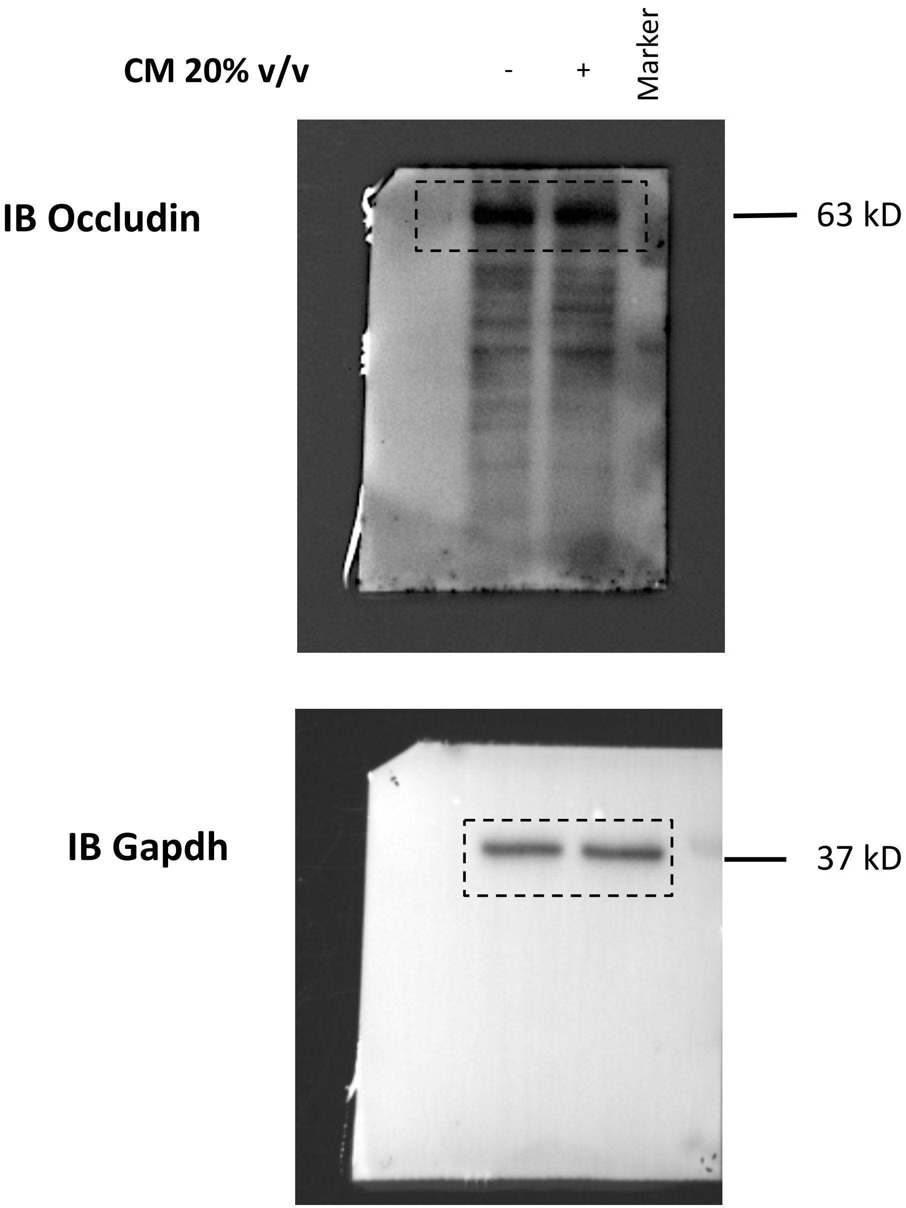

Figure S5

Full-length western blots images of cropped immunoblot of **Figure 5B**. Dashed squares correspond to the bands showed in the ultimate panels in the manuscript.

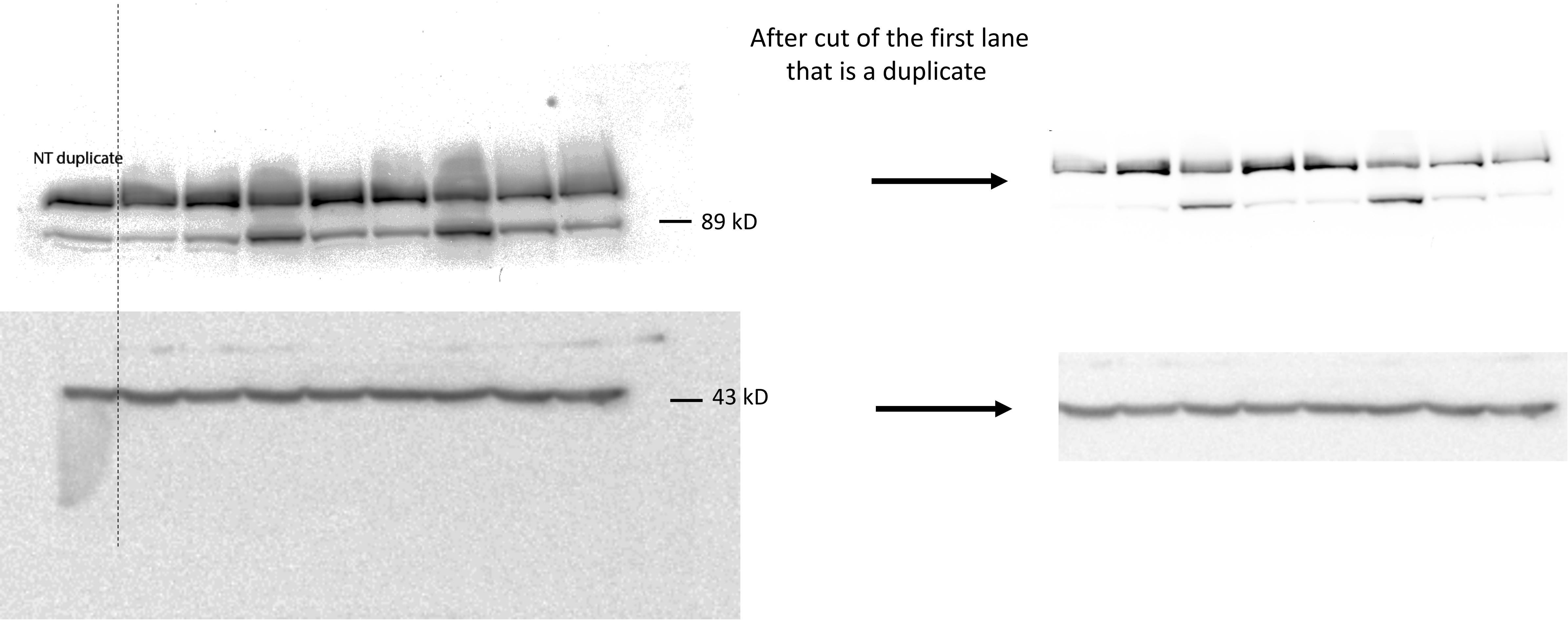

Figure S6

Full-length western blots images of cropped immunoblot of **Supplementary Figure 1 Part A**. Dashed squares correspond to the bands showed in the ultimate panels in the manuscript.

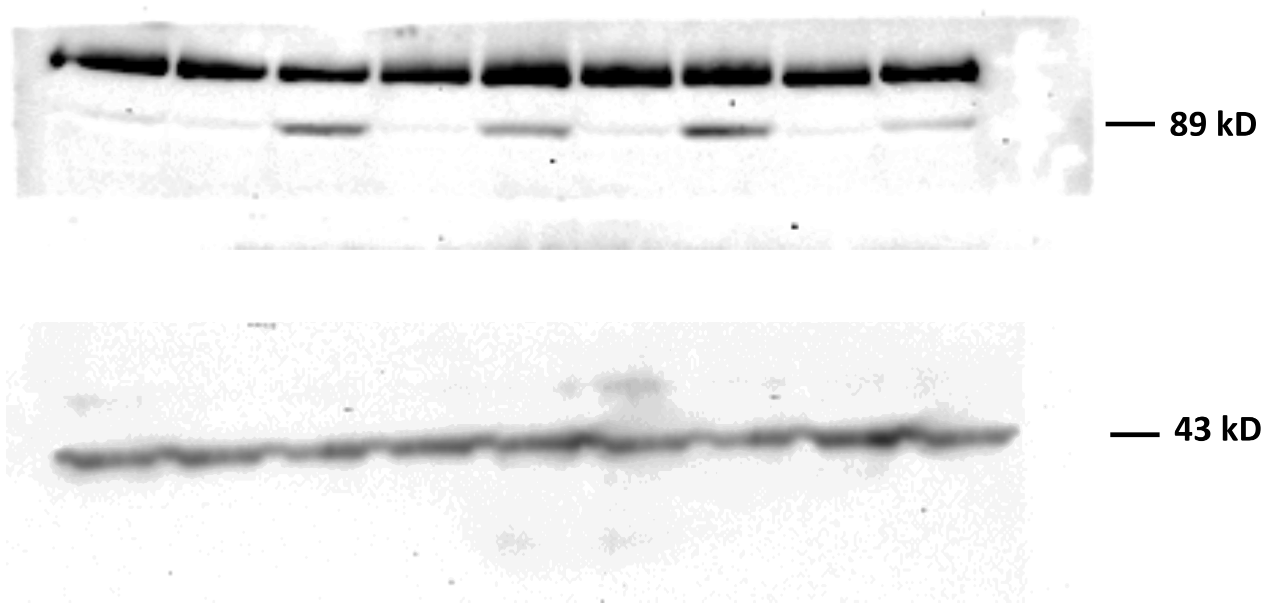

Figure S7

Full-length western blots images of cropped immunoblot of **Supplementary Figure 1 Part B**. Dashed squares correspond to the bands showed in the ultimate panels in the manuscript.
