# Supplementary material for: Modulation of intestinal epithelial cell proliferation and apoptosis by Lactobacillus gasseri SF1183
Source: Sci Rep. 2022 Nov 24;12:20248. doi: 10.1038/s41598-022-24483-0 (PMC9691729; doi:10.1038/s41598-022-24483-0)
Supplement: Supplementary file 2 — Supplementary Figure 1. [file 41598_2022_24483_MOESM2_ESM.pdf]

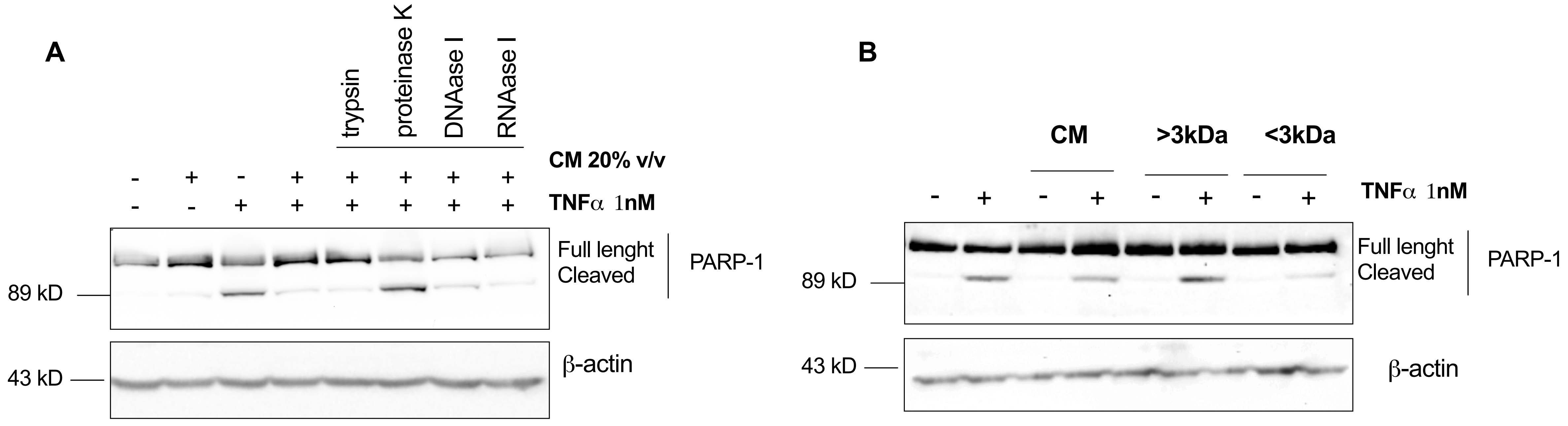

Conditioned Medium of *L.gasseri* SF1183 contains molecules smaller than 3kDa of proteinaceous nature.

HCT116 cells were pre-incubated for 16 hours with CM from *L. Gasseri SF1183* either untreated or

(A) treated with trypsin (GIBCO), proteinase K (Invitrogen), DNAase I or RNAase I (Invitrogen) at the final concentration of 100mg/ml for 60’ at 37°C

(B) size fractionated on a spin column (Centricon, Millipore) with a cut-off of 3 kDa

and then incubated or not with TNF-α 1nM for 8 hours. After the treatment cells were collected, lysed and total cell extracts were analyzed by western blot with anti-rabbit PARP-1 (Cell Signaling, EuroClone, Milan, Italy 9542) revaling both the full lenght and cleaved PARP-1 and β-actin (Santa Cruz) used as loading control.
